# Means-Inference as a Source of Variability in Early Helping

**DOI:** 10.3389/fpsyg.2018.01735

**Published:** 2018-09-26

**Authors:** Sophie Bridgers, Hyowon Gweon

**Affiliations:** Department of Psychology, Stanford University, Stanford, CA, United States

**Keywords:** prosocial behavior, instrumental helping, decision-making, causal reasoning, cost-benefit-analysis, cognitive development

## Abstract

Humans, as compared to their primate relatives, readily act on behalf of others: we help, inform, share resources with, and provide emotional comfort for others. Although these prosocial behaviors emerge early in life, some types of prosocial behaviors seem to emerge earlier than others, and some tasks elicit more reliable helping than others. Here we discuss existing perspectives on the sources of variability in early prosocial behaviors with a particular focus on the variability within the domain of instrumental helping. We suggest that successful helping behavior not only requires an understanding of others' goals (goal-inference), but also the ability to figure out *how* to help (means-inference). We review recent work that highlights two key factors that support means-inference: causal reasoning and sensitivity to the expected costs and rewards of actions. Once we begin to look closely at the process of deciding how to help someone, even a seemingly simple helping behavior is, in fact, a consequence of a sophisticated decision-making process; it involves reasoning about others (e.g., goals, actions, and beliefs), about the causal structure of the physical world, and about one's own ability to provide effective help. A finer-grained understanding of the role of these inferences may help explain the developmental trajectory of prosocial behaviors in early childhood. We discuss the promise of computational models that formalize this decision process and how this approach can provide additional insights into why humans show unparalleled propensity and flexibility in their ability to help others.

## 1. Introduction: variability in early prosocial behaviors

Humans are not only social creatures, we are also *pro*social. We often take actions that benefit others even at the expense of our own time, energy, and resources. The tendency to act on others' behalf emerges remarkably early in life; even preverbal infants readily help when others are struggling to achieve a goal (Warneken and Tomasello, 2006) or point to the locations of objects for which others are searching (Liszkowski et al., 2008). The fact that these behaviors emerge quite early in life has been taken as evidence for an intrinsic motivation to be helpful: We *want* to help others (Warneken and Tomasello, 2006; Tomasello, 2009).

Early prosocial behaviors have been categorized into different domains that vary in terms of “what” is being offered (Zahn-Waxler et al., 1992; Tomasello, 2009; Dunfield et al., 2011): Instrumental helping (physical, goal-directed action), informing (information), sharing (resources, such as food), and comforting (emotional expressions and gestures). Prior comparative and developmental work suggests that only some of these behaviors are shared with non-human primates while others may be uniquely human (Warneken, 1994; Warneken and Tomasello, 2009; Horner et al., 2011). Some work further suggests that even within humans, these behaviors may have rather independent developmental trajectories; helping and informing behaviors are observed at an earlier age than sharing or comforting (e.g., Liszkowski et al., 2008; Brownell et al., 2009; Svetlova et al., 2010), and children's tendency to act prosocially in one domain does not necessarily correlate with behaviors in other domains (Dunfield et al., 2011, 2013).

Such between-domain variability suggests that different prosocial behaviors may be subserved by different evolutionary roots and social-cognitive mechanisms (Tomasello, 2009). Researchers also appeal to different underlying motivational sources triggered by different cues (e.g., emotion contagion or emphathic concern triggers comforting, while goal-alignment leads to instrumental helping; Paulus, 2014). While these accounts may differ in their level of explanation and the proposed origins of differences observed across domains of prosocial behaviors, they generally agree that this variability reflects deeper differences between domains (e.g., Tomasello, 2009; Brownell, 2012; Dunfield et al., 2013; Paulus, 2014): We want to help *more* in some domains than others.

Wanting to help, however, is not the same as actually helping. For our prosocial motivation to lead to an action, it is also critical to figure out *how* to help. Depending on what others want, what went wrong, and what we can do to help, we may choose to help others in different ways, or not help at all. In fact, there is substantial variability even within a prosocial domain, raising the possibility that there may be other factors beyond between-domain differences in motivational sources and social-cognitive reasoning that influence children's tendency to help. However, the variability within domains has received relatively little attention.

Here we suggest that the pattern of data across different tasks within a domain can provide important insights into the development of prosocial behavior. We begin by taking a closer look at the variability in early instrumental helping in particular, and explore the nature of the inferences required by different tasks (i.e., inferences about others' goals and the means by which to help). We conclude by discussing how goals- and means-inferences can help explain the variability in early prosociality not only within but also across domains.

## 2. Variability in early instrumental helping: the role of goal-inference

**Figure 1:**
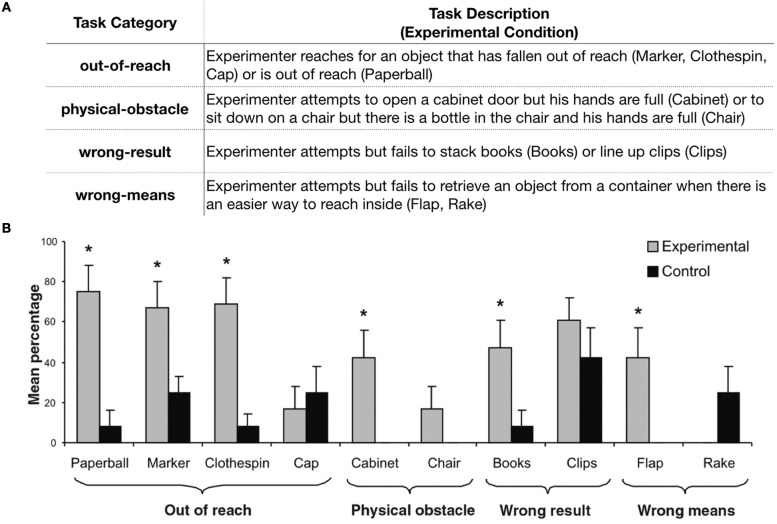
**(A)** A description of the different categories of instrumental helping tasks used in the experimental conditions of Warneken and Tomasello (2006); **(B)** Figure 1 from Warneken and Tomasello (2006) presenting the mean percentage of target behaviors for each task and condition. In the Paperball, Marker, Clothespin, Cabinet, Books, and Flap tasks, children performed the target behavior significantly more often in the experimental than in the control condition. No difference between conditions was found for the Cap, Chair, Clips, and Rake tasks. Error bars represent SE; ^*^p < 0.05. (Reproduced with permission from Warneken and Tomasello, 2006).

A seminal study by Warneken and Tomasello (2006) provides compelling evidence for the early emergence of instrumental helping. Human infants (18-month-olds) and chimpanzees were placed in a range of scenarios where a human adult attempted but failed to achieve a goal: (1) *out-of-reach* tasks, (2) *physical-obstacle* tasks, (3) *wrong-result* tasks, and (4) *wrong-means* tasks. (see Figure 1A for details). Both groups helped, though human infants did so more frequently and more reliably across different scenarios than chimpanzees. Based on these results, the authors argue that humans are naturally inclined to help, and that the motivation to help may have emerged sometime in evolutionary history before humans and chimpanzees diverged.

This study, however, also nicely demonstrates substantial within-domain variability in early instrumental helping behaviors. While often overlooked, these data are especially valuable because few studies have used such a wide range of tasks within the same domain (also see Warneken and Tomasello, 2007); most subsequent studies focused on comparing helping behaviors across domains and so used a subset of instrumental helping tasks that were shown to elicit high rates of helping. For our purposes, Warneken and Tomasello (2006) provides an ideal case-study for taking an in-depth look at the variability found in various instrumental helping tasks.

In this study, children most reliably helped in the out-of-reach tasks (over 60% in three of four tasks, see Figure 1B). They were more likely to pick up the out-of-reach object when the experimenter accidentally dropped and reached for it (experimental conditions) compared to when he intentionally threw it away and did not reach for it (control conditions), suggesting that they recognized the experimenter's goal and selectively provided help when he needed it. However, in other tasks children helped less frequently or not at all, or helped in both the experimental and control conditions. What might explain such variability?

One possibility, as the authors suggest, is that these tasks differ in how easy it is to infer the experimenter's goal from his behavior (Warneken and Tomasello, 2006). Yet, exactly how goal clarity might differ across tasks has not been explored in detail. Below we offer some speculation on the relationship between the difficulty of goal-inference and the rates of helping in this study.

The relatively lower rate of helping in some tasks can indeed be explained by goal ambiguity. In the Cabinet task (physical-obstacle), while the most plausible reason for why the experimenter is banging into the cabinet doors is that he wants to open them but his hands are full, he may have other reasons for doing so (e.g., trying to maneuver around the cabinet, or just doing it for fun). In contrast, the experimenter's goal may have been *too* clear in the Clips task (wrong-ends), eliciting the target action in both the experimental and control conditions. Here the experimenter has clips lined up on a board, and either unsuccessfully attempts to place three more clips on the board (experimental) or intentionally places the three clips *next to* the board (control). The already lined-up clips with three clips that remain off of the board might have led children to believe even in the control condition that the experimenter wanted them to place these remaining clips on the board.

In other tasks, however, the goal seems clear, yet children do not help reliably. For example, in the Rake task (wrong-means), the experimenter reaches for blocks inside of a vertical box with a transparent side, presumably making his goal as explicit as in the out-of-reach tasks. In the Chair task (physical-obstacle), the experimenter tries to sit down on a chair (but cannot because a bottle is on the seat), again making his goal of sitting down quite obvious. Nevertheless, both tasks elicited little to no instances of the target helping behavior. The absence of help is most striking in one of the out-of-reach tasks (Cap) where the experimenter reaches for his cap/hat, just like in other out-of-reach tasks. If the goal was clear in these tasks, why didn't children help? In order to help someone, the helper should of course understand what goal needs to be fulfilled (*goal-inference*). However, it takes more for a prosocial motivation to manifest as observable behavior; the helper also has to figure out *how* to help, or the *means* by which they can provide help. Below we discuss the role of this *means-inference* in more detail.

## 3. The importance of means-inference: figuring out *how* to help

*Means-inference* involves identifying the cause of the problem and the appropriate means to resolve the issue. Additionally, the helper needs to know whether this appropriate intervention is feasible and worthwhile to execute (i.e., costs and benefits of performing the action).

In the out-of-reach tasks in Warneken and Tomasello (2006), both goal- and means-inferences are relatively straightforward; the goal is clear, and the appropriate way to help is to simply re-enact the experimenter's action (i.e., reach to retrieve the object), which is well within preverbal infants' behavioral repertoire (Meltzoff, 1995; Hamlin et al., 2008).

However, comparing the tasks that elicited little helping (Rake, Chair, and Cap) with other tasks in the same category reveals how such inferences can be more complex. In the wrong-means Rake task, children watched an experimenter use the rake to retrieve objects but never used it themselves, whereas in the other wrong-means (Flap) task, children had a chance to lift the flap on the box before the critical test. Similarly, in the physical-obstacle Chair task, children did not have a chance to remove an object from the chair, whereas in the other physical-obstacle (Cabinet) task they were given an opportunity to open the cabinet doors. Finally, in the Cap task (the only out-of-reach task that did not elicit help), the out-of-reach object was the experimenter's personal possession, which might have made children unsure of whether it was okay to pick it up (i.e., permissibility of target action). These comparisons, while speculative, suggest that children's prior experience with the exact means to help may have influenced their tendency to help. It is difficult to say if children in this study could not infer the means, were uncertain about their ability (or the permissibility) to perform the means, or both. Nonetheless, these examples highlight that children's ability to understand how to help may impact whether they ultimately help even when the helpee's goal is clear.

Means-inference is often the crux of what makes helping hard. People in need of help are aware of their own goals, and often communicate these goals to the helper (e.g., “can you help me fix my computer?”), eliminating the need for goal-inference. However, figuring out how to help is usually up to the helper; most often, people need help because they do not know how to remedy their problem. Thus, studying the role of means-inference in particular might be critical to understanding what supports the planning and production of a helpful action, what might prevent us from producing it, and what changes across development.

Compared to goal-inference, means-inference has received comparatively less attention. Some studies discuss the difficulty of means-inference as a possible source of variability (e.g., Dunfield et al., 2011, 2013) but few directly investigate children's ability to infer the appropriate means to help while holding the goal and task constant. Below we discuss recent work that begins to shed light on young children's ability to figure out *how* to help.

### 3.1. Deciding how to help by identifying the cause of failure

One critical aspect of means-inference is a causal analysis of the situation: What is the source of the helpee's problem, and what can be done to address it? Depending on the cause of the helpee's failure, the helper may need to take different courses of action to resolve the problem. Prior work suggests that young children can make powerful and sophisticated causal inferences even from sparse evidence, aided by their understanding of others' knowledge, goals, and intentions (e.g., Gopnik et al., 2004; Kushnir et al., 2008; Shafto et al., 2012; Bridgers et al., 2016a; Sim et al., 2017). Remarkably, preverbal infants can infer the cause of their own failures based on the covariation information embedded in others' successes and failures (Gweon and Schulz, 2011). Yet, much remains unknown about how causal reasoning might inform how children help.

One recent study suggests that even toddlers readily recruit their causal knowledge to decide how to help (Bridgers et al., 2017). Two- and three-year-olds were introduced to three toys, each of which had a yellow button on one side that played music and a red inert button on the other side (but one of the toys was broken such that neither button played music). Then a naïve confederate pressed a button on one of the toys only to fail to play music and asked children for help. The only difference between the two conditions was whether the confederate tried the yellow button (suggesting that her toy was broken) or the red button (suggesting that she was trying the wrong side), but children responded in very different ways; they got her a different toy that worked in the first condition, but flipped the confederate's toy over to show her the correct (yellow) button in the second. The confederate's goal was very clear (she stated she wanted music), but knowing her goal was not enough: Knowledge of how the toys worked was critical to infer the source of the confederate's problem and select the appropriate means to help. These results suggest that even toddlers readily take advantage of what they have just learned minutes before to infer the correct means and provide effective help.

### 3.2. Deciding how to help via cost-benefit analyses of actions

Another critical aspect of means-inference is determining the feasibility of the means. This involves understanding whether one has the resources and the competence to perform the necessary action, and is socially permitted to do so. Recent work suggests that young children's tendency to help is affected by the expected difficulty of their own actions: Toddlers are less likely to offer instrumental help when it involves carrying a heavy object v. a light object, although their tendency to perform these actions increases as their motor capacity develops (Sommerville et al., 2018).

Beyond considering the physical costs of helping from their own perspective, children also begin to proactively consider the consequences of their actions for the helpee. When it is clear that obeying a specific request for help would not fulfill the helpee's goal (e.g., the requested cup has a crack), children override the request and help via a different means (e.g., giving her an intact cup; Martin and Olson, 2013). Given a forced choice, preschool-aged children also understand that it is more desirable to offer help with a difficult task than an easy one (Bridgers et al., 2016b; Bennett-Pierre et al., 2018). Furthermore, children are sensitive to whether reciprocity is encouraged and are more likely to help others who have engaged with them in reciprocal play than in parallel play (Barragan and Dweck, 2014). Children also become increasingly aware of the cultural normativity and permissibility of their own and others' actions (Nucci and Turiel, 1978; Rakoczy et al., 2008; Legare and Harris, 2016). The idea that objective and subjective costs of actions influence children's tendency to act prosocially is consistent with the proposal that humans have an intuitive understanding of the costs and rewards of their own and others' goal-directed actions (Gergely and Csibra, 2003; Jara-Ettinger et al., 2016).

Together, these studies suggest that helping is more than figuring out others' goals. It also involves recruiting one's knowledge to infer the appropriate means to resolve others' problems and determining whether it is feasible (or worthwhile) to help. If children are uncertain about any of these inferences, they may not help; not because they do not have the motivation or the desire to do so, but because they may be unsure of whether help is really needed, what actions make sense, or whether they are able to offer appropriate help.

### 3.3. Means-inference can give rise to different forms of prosocial behaviors

The significance of figuring out *how* to help might extend beyond instrumental helping. For example, in Bridgers et al. (2017), one can provide instrumental help to address the immediate cause of the confederate's failure, such as giving her one of the working toys or pressing the button that works. However, addressing the ultimate cause of the confederate's failure—her ignorance about how the toys work—would involve informing or teaching. Indeed, children's help was often accompanied by communicative behaviors that resemble pedagogical demonstrations (e.g., eye-contact, pointing; Csibra and Gergely, 2009) and even verbal information (e.g., “That one [toy] is not working”; “This button has no music.”), suggesting that some children were not only helping but also informing. Furthermore, if none of the confederate's toys played music, children might willingly *share* their own toy or even try to *comfort* the confederate to relieve her disappointment. The costs and feasibility of different means might also play a role; a child who wants to inform might resort to instrumental helping if her verbal proficiency is limited, or offer emotional comfort instead of giving away her favorite toy.

Our analysis illustrates how the lines between these different forms of helping may not be as distinct as previously proposed. Given the same goal, we can *choose* to provide instrumental help, information, resources, or comfort depending on the source of others' failure and what we can do to address it. Although there may be distinct perceptual, physiological, or cognitive mechanisms associated with different forms of prosocial behavior, their role might be to provide input to a more general decision-making process that generates the observed response^1^.

## 4. Prosocial behavior as a decision-making process

**Figure 2:**
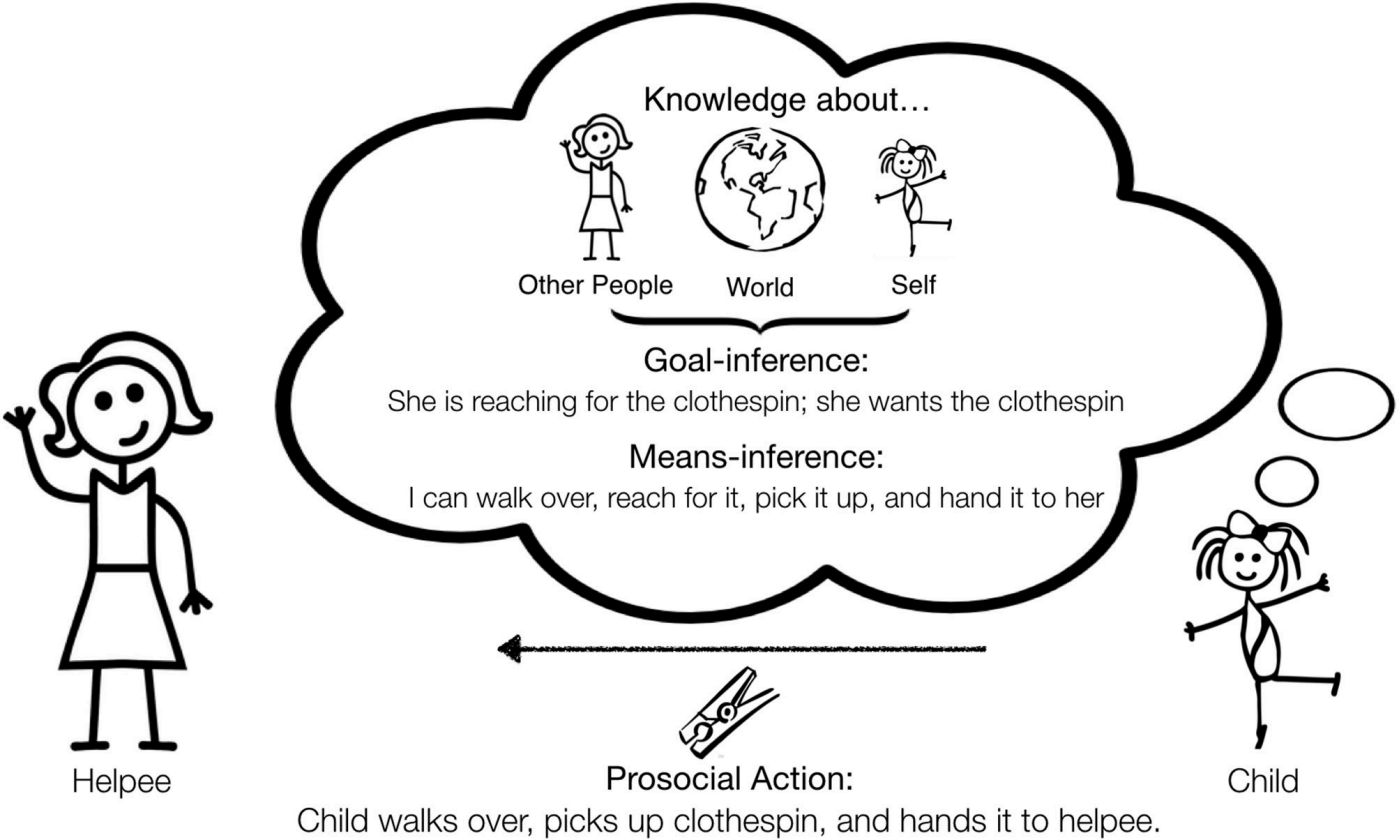
Schematic of the prosocial decision-making process. Goals- and means-inferences are critical to the decision to produce a prosocial action (instrumental helping in this case) and these inferences are influenced by reasoning about other people, the world, and the self.

A motivated helper needs to figure out what help is needed (goal-inference), what action would fulfill that need, and whether or not they are able to execute that action (means-inference). From this perspective, the production and form of prosocial behaviors are more than responses to cues or triggers; they are the output of a sophisticated *decision-making* process about the most effective way to help. We suggest that this process involves understanding the latent causal structure of the situation by integrating one's knowledge about (1) others (their goals, knowledge, preferences, competence, resources, etc.), (2) the physical world (intuitive physics, causality, etc.), and (3) the self (one's own knowledge, preferences, competence, resources, etc.) (see Figure 2). As children still have much to learn about each of these domains, investigating how children draw and coordinate inferences across these domains may help us better understand why some forms of helping seem to emerge earlier than others.

This framework highlights why it is difficult to draw strong developmental claims from the rates of helping across different tasks and situations alone. In studies that compare rates of helping across domains, the out-of-reach tasks are commonly used to measure children's tendency to help instrumentally (e.g., Svetlova et al., 2010; Dunfield et al., 2011, 2013). In these studies, younger children tend to instrumentally help more frequently than they share or comfort, which has led some authors to conclude that sharing and comforting involve more sophisticated social-reasoning and higher personal costs than instrumental helping. As our analysis reveals, however, children are less likely to provide instrumental help when the helpee's goal is ambiguous, means are hard to identify, or there is uncertainty about the feasibility of the needed actions. Even a strong desire to help may not produce an observable behavior if the appropriate means are unclear or the costs are too high (Jara-Ettinger et al., 2016). Thus, before concluding that competence in sharing and comforting emerges later in development (e.g., Brownell et al., 2009; Svetlova et al., 2010), it is important to ask if the tasks we use to index these abilities involve goals and means that are more ambiguous than the tasks we use to measure instrumental helping. It remains an open question whether tasks that are better matched along these dimensions would produce less variability across domains. Consistent with this possibility, in sharing tasks where the goal and means are made more explicit (e.g., the experimenter holds out her hand), children are more likely to share (Dunfield et al., 2011).

Our account does, however, motivate some clear developmental hypotheses. Young children may struggle to infer the helpee's goal or figure out the means to help, or may lack the necessary competence or resources to help; thus with increased age and experience, the frequency of helping, as well as the diversity and sophistication of the means employed may increase. Furthermore, as children's reasoning about others' minds develops across early childhood, they may become better able to signal their helpful intent regardless of the effectiveness of their actions, and even begin to show adult-like sensitivity to how their help might be perceived by the helpee (e.g., seeing helping as patronizing, etc.).

A key challenge in drawing developmental conclusions from behavioral data is that the absence of a particular behavior does not entail the absence of underlying mental constructs (e.g., motivation to help, the ability to draw goal- and means-inferences, physical competence, etc.). Computational models can complement developmental methods because they are particularly useful in revealing how multiple decision factors interact and contribute to generating behavior (see Shafto et al., 2014; Jara-Ettinger et al., 2016, for recent computational work on social cognition). Characterizing prosocial behavior as the output of a decision-making process lends itself well to formalization (Tenenbaum et al., 2011; Berger, 2013). This approach would force researchers to express the inferences involved (and the knowledge that supports them) in precise, quantitative terms, and would generate graded predictions about how likely children are to help, what form this help might take, and how effective it is likely to be in a given situation.

## 5. Concluding thoughts

In sum, while prior work has found compelling evidence for a remarkably early emergence of prosocial behavior, it also has found substantial within-domain variability across different kinds of instrumental helping tasks. Because the primary question in previous studies was whether preverbal infants *can* help at all, this variability has not received as much attention as variability across domains, and was largely attributed to children's developing ability to identify others' goals.

However, a closer look at different tasks raises the possibility that the ease of figuring out *how* to help may also modulate children's tendency to help. Studying early prosocial behavior as a decision-making process highlights the importance of both goals- and means-inferences, provides grounds for connecting developmental literature with studies of cooperative behaviors in adults (e.g., Rand and Nowak, 2013), and opens up avenues for computational research investigating how intuitive theories and inferential abilities allow prosocial motivations to manifest as observable, effective actions. Our analysis also highlights the importance of taking seriously the inferential demands of different tasks; by designing tasks that systematically vary in the complexity of the goal- and means-inferences involved, we can better characterize children's helping abilities both within and across domains.

Humans are not only motivated to help; we are also good at it. Studying how children become able helpers, knowledgeable teachers, effective cooperators, and empathic companions will allow us to better understand how across generations humans have accomplished so much and become the most powerful and flexible species on the planet.

## Author contributions

All authors listed have made a substantial, direct and intellectual contribution to the work, and approved it for publication.

### Conflict of interest statement

The authors declare that the research was conducted in the absence of any commercial or financial relationships that could be construed as a potential conflict of interest.

## Footnotes

^1^While some prosocial behaviors might be driven by immediate physiological responses to others' plight or danger, most laboratory studies are likely probing more deliberate decisions to help. Here, we constrain our focus to how we can better evaluate and compare these different experimental tasks.
